# Attentional bias in paranoia: systematic review and meta-analysis

**DOI:** 10.1192/bjo.2026.10993

**Published:** 2026-04-06

**Authors:** Laura Eid, Chloe Hampshire, George Vamvakas, Pamela Jacobsen, Daniel Stahl, Jenny Yiend

**Affiliations:** Department of Psychosis Studies, Institute of Psychiatry, Psychology and Neuroscience, King’s College London, UK; Bath Centre for Mindfulness and Compassion, Addiction and Mental Health Group, Department of Psychology, University of Bath, UK; Department of Biostatistics and Health Informatics, Institute of Psychiatry, Psychology and Neuroscience, King’s College London, UK

**Keywords:** Psychosis, cognition, attention bias, threat, paranoia, systematic review, meta-analysis

## Abstract

**Background:**

Paranoia is a transdiagnostic symptom and is associated with cognitive and social impairments. Attentional bias toward threat is thought to maintain paranoia.

**Aims:**

Despite many studies, attentional biases in paranoia have not been systematically summarised, which was the aim of the current work.

**Method:**

We conducted a systematic review and meta-analysis, identifying 10 964 studies, of which 35 met inclusion criteria for review and 15 for meta-analysis.

**Results:**

Findings showed a significant negative attentional bias (average standardised effect size 0.26; 95% CI 0.01–0.52; *p* = 0.046). Preliminary indications suggested bias was strongest for paranoia-related stimuli (average effect size 0.30; 95% CI 0.03–0.57; *p* = 0.027) and stronger for words than faces (average effect size 0.41; 95% CI 0.05–0.77; *p* = 0.027), but more data is needed to confirm these effects. Limitations were primarily statistical and included likely underestimation of the overall effect size of the association between negative attentional bias and paranoia and a lack of sufficient studies to robustly examine moderators.

**Conclusions:**

Summarising this literature provides a rationale for existing and new interventions for paranoia that target biased attentional mechanisms.

Attentional biases are thought to exacerbate and maintain the symptoms of a wide variety of psychological disorders, including psychosis.^1,2^ Cognitive models of psychosis and paranoia suggest that attentional bias (along with other biases) are both causal and perpetuating factors for persecutory delusions.^3,4^ An attentional bias occurs when one particular type of information in the environment is selected for processing over and above everything else that could be attended to. For example, some evidence suggests that anxious individuals have a slower response time to naming the colour of emotional words in Stroop tasks, suggesting an attentional bias to threat.^5–7^ Similar findings are apparent in depression^8,9^ and eating disorders,^10,11^ to name just a few further psychopathologies. When attentional bias occurs in the context of a psychological disorder, the information that is selected for priority processing usually matches the core symptoms and concerns associated with the disorder,^5^ a concept referred as content specificity. For example, a higher degree of content specificity is demonstrated in individuals with high trait paranoia and those with clinical paranoia who show a heightened interpretation bias toward paranoia-related items compared with other ambiguous material.^12,13^

There is substantial research literature on the attentional biases associated with paranoia and psychosis. Studies of individuals experiencing persecutory delusions find Stroop interference for affective^14^ and paranoia-related words.^15^ In schizophrenia, the pattern is similar where individuals with a schizophrenia diagnosis respond faster to threat-related stimuli.^16^ Reviews have been conducted on a mixture of different cognitive biases in psychosis,^17–19^ reasoning biases in psychosis and delusions,^20^ and perception of emotional faces in schizophrenia.^21–23^ However, no review or meta-analysis has been published to date specifically on attentional biases associated with paranoia. Indeed, within this, there is noticeable variation in the findings reported. For instance, Combs and Penn^24^ reported stronger attention bias to paranoia and psychosis-related words in individuals with high subclinical paranoia, a finding different from the results in Wiffen et al.^25^ By quantitatively summarising the literature in the field and examining possible moderators, the present review will help identify how between-study variance can best be explained.

An important distinction has been made between two different levels of contrast that can reflect attention bias.^26^ The first level is the ‘within participant bias’, which refers to the difference in bias at the participant level between attention to different types of stimuli (e.g. emotional versus neutral). The second, is the ‘between participant bias’, defined as the difference in the direction and/or magnitude of attention bias between healthy controls versus individuals with a psychopathology. For example, individuals with psychosis may demonstrate a within-participant attention bias by selecting emotional stimuli for processing over and above neutral. A between-participant attention bias would be evidenced where a study reported a difference between the psychosis sample and a control sample in the magnitude and/or direction of selective attention across emotional and neutral information. For the purpose of this systematic review, both levels of bias evidence were included.

We aimed to conduct a systematic review with meta-analysis to provide an overall conceptualisation of findings from the field, taking into account individual studies’ differences in quality, sample size and related methodological differences. We planned to use meta-regression as a tool to identify any study specific systematic factors that could account for some of the variation in effect sizes observed across clusters of studies. The attentional bias literature suggests a number of *a priori* conceptual and methodological factors that might be expected to influence the pattern of data observed in any given study. For the present review, we identified the following four areas of potential interest.

The first potential moderator of results we considered was the clinical or subclinical status of the sample. Given that biases are maintaining features of anxiety and paranoia,^2^ one would expect that attention bias differs according to clinical status. There are at least two reasons why one would expect to see differences. First, those with a clinical diagnosis of psychosis (i.e. clinical status) will typically display more severe symptoms than those reporting subthreshold symptoms (i.e. subclinical status). In other disorders, greater symptom severity levels has been associated with stronger attentional bias,^27^ and one might therefore expect the same in paranoia. Second, some authors have suggested that there could be qualitative differences between clinical and subclinical status, irrespective of actual symptom levels (e.g. Schreuder et al).^28^ To our knowledge, no study explicitly examined the relationship between attention bias and clinical status/symptom severity in psychosis or paranoia. By including clinical status as a moderator, the current review will address this gap in the literature.

The second potential moderator was content specificity (stronger bias for material that matches the primary concerns of the disorder) as a feature of interest across the attention bias literature. Some studies suggest that a disorder-congruent threat bias exists in anxiety disorder.^29^ The range of emotions typically studied in paranoia and psychosis include sadness, anger and fear, with a few including paranoia-specific material. Research on attention bias specificity has revealed mixed findings, highlighting the importance of the current systematic review in understanding what moderates attention bias to threat in people with paranoia. For example, evidence from a previous study by Besnier et al^30^ suggested an attention bias to words related to paranoia and psychosis-related stimuli in people with paranoia and schizophrenia, but no bias to depression-related stimuli.

Type of attentional task was the third moderator of interest. A wide range of methods have been developed to measure attention bias, which tap into different aspects of attention,^31^ including (but not limited to) the emotional Stroop task,^32^ the emotional attentional probe task,^33^ the visual search task^34^ and the spatial cueing task.^35^ Here we introduce the two most common tasks, the emotional Stroop and attentional probe task. The emotional Stroop requires participants to name the colour of neutral and emotional words and measures attentional bias by calculating an interference index, which reflects the additional time taken for participants to name the colour of the emotional words compared with neutral words. A bias in favour of emotional material is reflected by reaction time slowing. In the attentional probe task bias is also measured by looking at response time differences between emotional and neutral stimuli, but now bias in favour of emotion material is reflected by reaction time speeding. Individuals displaying a bias toward emotion respond faster when probes replace emotional material, compared with neutral material, because their attention is already located at the emotion location when the probe appears here, but has to shift when the probe appears elsewhere. It is important for the research field to know whether any particular tasks are more or less sensitive to detect attentional biases in this population, as this could usefully inform future research designs.

Our final moderator of interest was the type of stimuli attended, faces or words, as both have been used extensively in the literature. Each is known to engage different areas of the brain in early-stage processing, meaning that connectivity with attentional areas will differ and the impact on attentional bias may differ correspondingly. Furthermore, as people with paranoia are likely to spend longer thinking and talking about threat, threat-related words may be primed relative to face stimuli, suggesting word tasks may reveal a larger bias.^5^ However, facial expression is inherently social, and social threat is a core part of paranoia. Indeed, social evaluative concerns are the most common type of suspicious thoughts and appear at the start of Freeman’s paranoia hierarchy.^36^ This suggests that faces, especially if angry or fearful, could be powerful elicitors of negative attentional bias for individuals with paranoia. For example, delusion-prone individuals showed a stronger attentional bias to angry faces compared with happy faces,^37^ and patients with persecutory delusions demonstrated a stronger attentional bias to paranoia-related words compared with neutral words.^15^ To our knowledge, no study directly compares the strength of attentional bias when using facial versus and word stimuli in paranoia. The current meta-analysis therefore provides a good opportunity to investigate if there are any systematic differences in attention bias in paranoia related to this stimulus type.

A potential moderator initially considered was the duration of the stimulus presented. The rationale for studying subliminal stimuli is to explore the extent to which an automatic, unconscious processing of threat is more apparent in individuals with paranoia, as this feature has found to be important in other areas of the bias literature. This variable was considered in the systematic review, but could not be included as a moderator in the meta-analysis because of a lack of studies reporting subliminal data.

In summary, our review aimed to quantify the strength of the association between paranoia and negative attentional bias, and examine potential moderators to help better understand the overall picture from the literature to date. We addressed the following questions:

- Is paranoia associated with a selective attentional bias toward negative compared with neutral information? (Analysis 1)
- Does any association found apply in both clinical and subclinical groups? (Analysis 2)
- Does any association vary according to stimulus emotional content? (Analysis 3)
- Does paranoia-related attention bias vary according to the task used to measure it? (Analysis 4)
- Does the strength of association vary across facial and word stimuli? (Analysis 5)

## Method

### Transparency and openness

#### Pre-registration

The review protocol was pre-registered on the open-access PROSPERO website (reference CRD42022291794) on 9 February 2022, before searches were conducted. Date of initial extraction was 11 March 2022 (updated on 1 May 2025).

#### Data, materials, code and online resources

Code used to perform the analyses is available at the following link: https://osf.io/t9ayn/. The Supplementary Materials contain two files, one with further details of the statistical analyses and a second with further details of the risk-of-bias assessment and results.

#### Reporting

This study involved an analysis of existing data rather than new data collection. We confirm that we have reported all analyses conducted as part of this work.

#### Ethical approval

Ethical approval was not required as analyses were conducted on fully anonymised aggregated data only, which was already available in the public domain.

### Search strategy

**Fig. 1:**
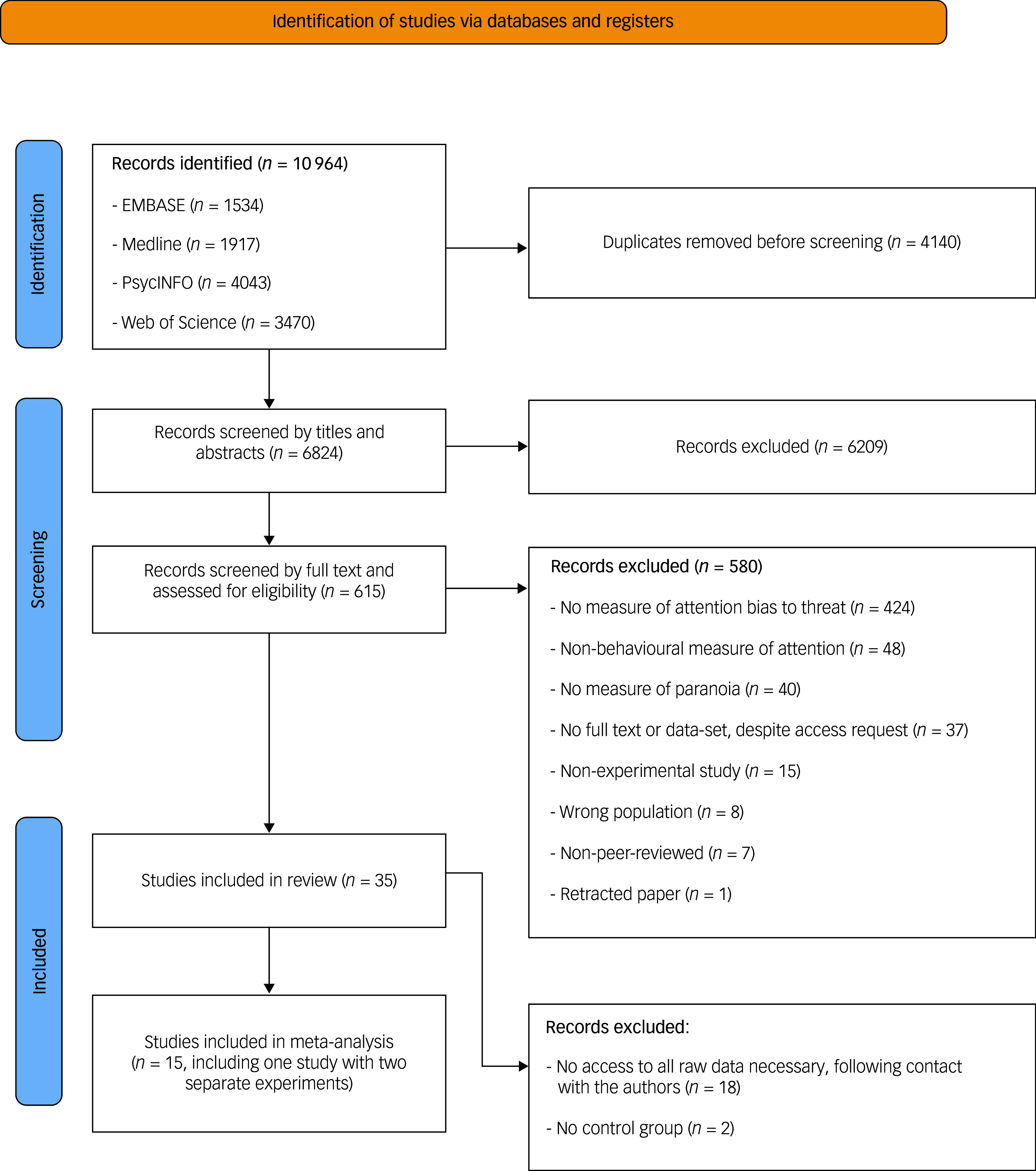
Preferred reporting items for systematic reviews and meta-analyses^38^ flowchart describing the selection process.

A systematic search was conducted in 15 March 2022, using the following databases: Web of Science, EMBASE, Medline and PsycINFO. The search terms were (psychos* OR paranoi* OR manic OR mania OR schizo* Or psychotic OR delusion*) AND (attention* bias* OR dot-probe OR Stroop OR flanker OR gaze direct* bias OR posner OR visual attention OR attention* process* OR salien* attention* or cognit* bias* OR bias* modifi* or selective attention OR attention* disengagement OR visual explor* OR spatial cue OR visual scan* OR visual search*). If available, filters were added to limit the year range (1980 onward), to include only peer-reviewed papers and exclude studies on animals. The search strategy is summarised in Fig. 1 below. The search was updated on 1 May 2025, identifying two papers that met the inclusion and exclusion criteria; however, neither provided the required data, and attempts to contact the authors for access were unsuccessful.

### Inclusion criteria

We included behavioural studies of attention bias, where bias was conceptualised by the authors as selective attention to emotional stimuli in preference to designated control stimuli (e.g. neutral). Following De Lissnyder et al,^26^ the contrast between the emotional stimulus and the control stimulus was designated as the ‘within-participants contrast’.

Attention bias was measured in adult or mixed-age span (samples including both adults and children, without separating the groups) patients in one of the following groups:

- patients with a clinical diagnosis of psychosis/schizophrenia spectrum disorder, OR
- individuals experiencing symptoms of paranoia that reach a clinical threshold according to a validated self-report or clinician-administered measure OR
- individuals with subclinical paranoid symptoms according to a validated self-report or clinician-administered measure.

We also included trials or intervention studies in which an attentional bias to emotional information paradigm was used on individuals with paranoia, including but not limited to, visual search studies, attentional probe task, emotional Stroop tasks, emotional spatial cueing tasks and continuous performance tasks.

Finally, we included studies that used a control group, defined as either

- a within-participants control (i.e. an internal contrast between an emotional and a control stimulus) or
- a between-participants control defined as participants with no current clinical or subclinical psychopathology.^26^

### Exclusion criteria

We excluded studies that included psychosis comorbid with another disorder; studies that only reported physiological or neurobiological measures of attention (e.g. eye-tracking or neuroimaging); samples involving children only; case reports, reviews and non-peer-reviewed literature; and studies published before 1980.

### Study selection

Covidence (a web-based software aimed at organising and streamlining the processes involved in the systematic reviews, including screening of records and data extraction) was used to conduct the review (Covidence systematic review software, Veritas Health Innovation, Melbourne, Australia; available at www.covidence.org).

Duplicate studies were removed (automatically by Covidence and manually where necessary). The remaining studies were screened by titles and abstracts, against the inclusion and exclusion criteria. Potentially eligible studies progressed to the full-text screening stage. Where the full text was not accessible, authors were contacted. All records were independently double-screened by two reviewers (L.E. and C.H.) at all stages, including the title and abstract screening and full-text screening. Any resulting discrepancies were resolved by discussion among the raters to reach a consensus, and by consultation with the senior authors (J.Y.), whenever required. Interrater reliability between the two reviewers was 0.89 for the title and abstract screening and 0.96 for the full-text screening.

### Data extraction

Data extraction was done independently by two reviewers (L.E. and C.H.) using a prespecified data extraction spreadsheet approved by all authors. Where the raw data or data necessary to calculate interference indices or attentional bias score was missing, authors were contacted to try to obtain these data. The extracted independent variable data included title, authors, country, year of publication, total sample size, sample size by group, age and standard deviation of each group.

A limitation of our extraction process was that following demographic data were not extracted: racial/ethnic identification, sex/gender, income, education and socioeconomic status. Preliminary searches indicated the final sample size of included papers would be insufficient to allow examination, in meta-analyses, of any variables beyond those central to our hypotheses. However the narrative review could have incorporated these demographics, but did not for reasons of time and feasibility of the data extraction.

The outcome data extracted were either mean raw data for emotional and control conditions, or authors’ own derived attentional bias scores. The latter were used if the primary condition mean data were not available after contacting the authors. All subsets of data of potential relevance to the five research questions were also extracted. This included separate means for all conditions related to clinical grouping, stimulus emotional content, attentional task type and facial or word stimuli.

### Data synthesis

As a standardised effect size, Hedges’ *g*, a variation of Cohen’s *d*, was used. Similar to Cohen’s *d*, it is calculated by dividing the difference in means between two groups by the pooled standard deviation, and is then corrected by a small sample size bias correction. For all studies, attention bias mean and standard deviation for both experimental and control groups, separately, were calculated by using the raw mean and standard deviation of reaction times to calculate the within-participant bias (the difference in time needed for an individual with paranoia to attend to an emotional stimulus compared with a neutral one). All bias score calculations were conducted such that a more positive value reflected a stronger bias toward the emotion compared with the neutral material. For example, emotional Stroop bias scores were calculated by subtracting the neutral from the emotion reaction time because bias favouring emotion is captured in this task when reaction times to colour name emotion words are slowed. In contrast, attention-probe bias score was calculated by the opposite subtraction (probe detection speed on neutral-probed minus emotion-probed trials), because now bias favouring emotion is captured by speeding to detect probes replacing this material, compared with neutral material. In all instances, a larger bias score signified greater attention toward the emotion. Attention bias standard deviations were calculated using the pooled standard deviation formula to ensure a consistent calculation for all studies. Calculation of between-participant biases (i.e. mean differences between experimental and control groups) were calculated at the analysis stage, as described below.

### Risk of bias

Risk-of-bias assessments were conducted independently by two reviewers (L.E. and C.H.) for each included study, using the Effective Public Health Practice Project (EPHPP) tool.^39^ The EPHPP consists of eight components, six of which determine the overall quality of the study. A supplementary dictionary was available to describe each component to aid raters to evaluate the quality of the studies in the most homogenous manner.

### Statistical analysis

Studies included in the meta-analysis consisted of all papers included in the systematic review that reported the data necessary to calculate attentional bias scores. Key features of the analysis are reported here, with full details provided in Supplementary File 1. Throughout the paper, statistical significance was assessed at the 5% level. The meta-analyses were performed by pooling the standard effect sizes, using a random-effects model. Meta-regression models with robust standard errors were used to analyse the outcomes and allow for effect size dependencies that emerge from the inclusion of studies from the same research groups.^40^ The *τ* ^2^ and the *I* ^2^ statistics are presented for each meta-regression model to express the between-cluster variance (*τ* ^2^) and the amount of variability in the effect size estimates due to effect size heterogeneity as opposed to random variation (*I* ^2^). Analyses were carried out in Stata version 17, using Robumeta for meta-analyses and meta-regression (Hedberg 2011).^41^ The *τ* ^2^ and the *I* ^2^ statistics are presented for each meta-regression model to express the between-cluster variance (*τ* ^2^) and the amount of variability in the effect size estimates due to effect size heterogeneity as opposed to random variation (*I* ^2^).

#### Sensitivity analyses

Some of the analyses reported here involved only a small number of studies. When estimation includes a small number of studies (fewer than four Satterthwaite degrees of freedom) and/or covariates with high leverage, this can result in inflated type one errors (see Tipton^38^). In this case, Tipton^38^ recommends re-running the analysis after removing unusual or influential data points. We therefore performed sensitivity analyses, involving a sequential process of re-running the analysis after leaving out one effect size at a time and checking whether the exclusion of the effect size materially altered the inference. The results from these sensitivity analyses are reported alongside the main results of each analysis in the form of the minimum and maximum coefficients returned after concluding all iterations (Supplementary File 1). This permits the reader to judge the reliability of any findings arising from fewer than five studies.

#### Analysis outline

We conducted five analyses, corresponding to each of our main research questions. Each one adjusting the standardised attention bias effect size for a different set of moderator variables. The first analysis included data relating to negative emotional stimuli only and addressed the overarching research question (question a), whether paranoia was associated with a selective attentional bias toward negative rather than neutral information. The same data were used to address questions b, d and e. To address question c (differences according to stimulus emotional content), data relating to positive stimuli were added into the model. Further analytical details are provided in Supplementary File 1.

## Results

### Systematic review

Overall, 10 964 studies were initially identified through database searching.

After removing duplicates (*n* = 4140), 6823 titles and abstracts were screened, resulting in 6209 exclusions against the criteria. This left 617 articles for full -ext screening, leading to a further 582 exclusions. Reasons for exclusion included: no measure of attention bias to threat, no behavioural measure of attention, no measure of paranoia, no full text despite access request, non-experimental study, wrong target population, non-peer-reviewed article or retracted paper. The final systematic review comprised 35 studies, of which 15 had adequate quantitative data for inclusion in the meta-analysis. Figure 1 illustrates a detailed flow chart of the Preferred Reporting Items for Systematic Reviews and Meta-Analyses study selection process.^42^ The studies included in the systematic review are summarised in Table 1.

**Table 1:** Systematic review study characteristics

Author, year	Country	*N* total	Subgroups (clinical status): *n*	Subgroups: age, years, mean (s.d.)	Experimental paradigm	Stimuli, type	Content specificity	Duration of stimulus presentation
^a^Aleksandrowicz et al, 2020^43^	Switzerland	135	High risk (clinical): 41 Ultra high risk (clinical): 48 Healthy controls: 46	High risk: 23.6 (5.6) Ultra high risk: 18.3 (3.5) Healthy controls: 21.2 (5.3)	Emotional Stroop task	Visual, words, supraliminal	Neutral, negative or positive	1500 ms
Andersen et al, 2016^44^	USA	78	Schizophrenia (clinical): 31 Healthy controls: 47	Schizophrenia: 24.8 (5.2) Healthy controls: 26.8 (7.0)	Emotional oddball task	Visual, images, supraliminal	Standard, neutral, aversive or target (non-threatening)	500 ms
^a^Arguedas et al, 2006^37^	Australia	44	High PDI (subclinical): 22 Low PDI (subclinical): 22	High PDI: 21.78 (7.6) Low PDI: 24.14 (9.05)	Attentional probe task	Visual, faces, supraliminal	Neutral, angry or happy	–
Barbalat et al, 2012^45^	UK	36	Schizophrenia (clinical): 18 Healthy controls: 18	Schizophrenia: 38.4 (7.7) Healthy controls: 35.5 (7.8)	Emotion discrimination task	Visual, faces, supraliminal	Happy, fearful, angry or neutral	1500 ms
Bendall et al, 2014^46^	Australia	67	FEP (clinical): 42 Healthy controls: 25	Healthy controls: 21.12 (2.46)	Emotional Stroop task	Visual, words, supraliminal	Positive, negative, threat or neutral	1500 ms
^a^Bentall and Kaney, 1989^15^	UK	32	Schizophrenia/delusional disorder (clinical): 16 Healthy controls: 16	Schizophrenia/delusional disorder: 32.93 (12.86) Healthy controls: 32.94 (10.46)	Emotional Stroop task	Visual, words, supraliminal	Os, neutral, depressive or paranoid	–
^a^Besnier et al, 2011^30^	France	90	Paranoid schizophrenia (clinical): 30 Healthy controls: 60	Paranoid schizophrenia: 33.5 (7.69) Healthy controls: 38.53 (11.44)	Emotional Stroop task	Visual, words, supraliminal	Xs, neutral, paranoid, depressive, manic	–
Caruana et al, 2021^47^	Australia	38	Schizophrenia/schizoaffective disorder (clinical): 18 Healthy controls: 20	Schizophrenia/schizoaffective disorder: 53.56 (7.66) Healthy controls: 50.80 (11.99)	Continuous flash suppression	Visual, faces, subliminal	Anger, fear	100 ms
^a^Combs and Penn, 2004^24^	USA	60	High paranoia (subclinical): 29 Low paranoia (subclinical): 31	High paranoia: 20.3 (3.4) Low paranoia: 21.2 (2.3)	Emotional Stroop task	Visual, words, supraliminal	Non-threatening, paranoia or depression	–
^a^Demily et al, 2010^48^	France	41	Schizophrenia (clinical): 21 Healthy controls: 20	Schizphrenia: 33.1 (8.8) Healthy controls: 31.8 (6.2)	Emotional Stroop task	Visual, words, supraliminal	Positive, negative or neutral	2500 ms
Fear et al, 1996^49^	UK	28	Delusional disorder (clinical): 28	Delusional disorder: 51.45 (15.43)	Emotional Stroop task	Visual, words, supraliminal	Os, neutral, depression, threat or anxiety	–
^a^Fear et al, 1996^49^	UK	49	Delusional disorder (clinical): 29 Healthy controls: 20	Delusional disorder: 52 (14.6) Healthy controls: –	Emotional Stroop task	Visual, words, supraliminal	Os, neutral, depression, threat or anxiety	–
Feroz et al, 2019^50^	Germany	40	Schizophrenia (clinical): 20 Healthy controls: 20	Schizophrenia: 32.65 (9.78) Healthy controls: 32 (9.38)	Emotional Stroop task	Visual, words, supraliminal	Positive, negative or neutral	–
^a^Holper et al, 2016^51^	Switzerland	174	Paranoia/psychoticism (subclinical): 147 Healthy controls: 27	Paranoia: 29.64 (6.69) Healthy controls: 29.86 (5.78)	Emotional Stroop task	Visual, words, supraliminal	Positive, negative or neutral	1500 ms
Hurtado et al, 2018^52^	Spain	37	Schizophrenia (clinical): 19 Healthy controls: 18	Schizophrenia: 34.05 (8.78) Healthy controls: 33.89 (12.03)	Modified Egner’s face task – emotional version	visual – faces and words, supraliminal	Happiness, fear	2000 ms
Ilankovic et al, 2011^53^	Germany	46	Paranoid schizophrenia (clinical): 23 Healthy controls: 23	Paranoid schizophrenia: 33.26 (9.3) Healthy controls: 33.78 (9.26)	Audio–visual experimental task	Audio–visual, voice and faces, supraliminal	Self (realistic and distorted), alien (alien voice or distorted)	Picture presented for 200 ms, followed by the voice
^a^Jang et al, 2016^54^	South Korea	41	Schizophrenia/schizoaffective disorder (clinical): 22 Healthy controls: 19	Schizophrenia/schizoaffective disorder: 41.91 (8.09) Healthy controls: 41.74 (8.03)	Attentional probe task	Visual, faces, supraliminal	Happiness, sadness, anger	500 ms or 1500 ms
^a^Jang et al, 2016^55^	South Korea	51	Schizophrenia (clinical): 30 Healthy controls: 21	Schizophrenia: 41.23 (10.12) Healthy controls: 36.38 (5.15)	Attentional probe task	Visual, faces, supraliminal	Happiness, sadness, anger	500 ms or 1000 ms
^a^Kinderman et al, 1994^56^	UK	32	Schizophrenia/delusional disorder (clinical): 16 Healthy controls: 16	Schizophrenia/delusional disorder: 34.3 (12.5) Healthy controls: 31.3 (11)	Emotional Stroop task	Visual, words, supraliminal	Os, neutral, positive or negative	–
Kinderman et al, 2003^57^	UK	26	Persecutory delusions (clinical): 13 Healthy controls: 13	–	Emotional Stroop task	Visual, words, supraliminal	Threat (sociotropy, autonomy, physical, ego – other, self-directed ego)	–
Klewchuk et al, 2007^58^	UK	18	Schizophrenia (clinical): 18	Schizophrenia: –	Emotional Stroop task	Visual, words, supraliminal	Negative, positive or neutral	–
Lim et al, 2011^59^	Australia	88	FEP/chronic psychoticism (clinical): 25 Healthy controls: 63	FEP/chronic psychoticism: 24.6 (6.08) Healthy controls: 24 (6.29)	Emotional Stroop task	Visual, words, supraliminal	Threat, positive, depressive or neutral	1500 ms
Liu et al, 2016^60^	China	53	Schizophrenia (clinical): 27 Healthy controls: 26	Schizophrenia: 21.6 Healthy controls: 23.2	Modified flanker task	Visual, faces, subliminal	Fear or neutral	100 ms
^a^Marley et al, 2017^61^	UK	52	Paranoid schizophrenia (clinical): 32 Healthy controls: 20	Paranoid schizophrenia: 35.48 (7.78) Healthy controls: 35.55 (5.24)	Attentional probe task	Visual, words, supraliminal	‘Poor me’, ‘bad me’ or neutral	–
Moritz et al, 2007^62^	Germany	58	Schizophrenia (clinical): 24 Healthy controls: 34	Schizophrenia: 35.21 (11.29) Healthy controls: 35.85 (13.23)	Attentional probe task	Visual, images, supraliminal	Paranoia, anxiety or neutral	400 ms
Penn et al, 1994^63^	USA	38	Schizophrenia/schizoaffective disorder (clinical): 38	Schizophrenia/schizoaffective disorder: 36.2	Emotional Stroop task	Visual, words, supraliminal	Social threat, physical threat, Xs or neutral	–
Pinkham et al, 2014^64^	USA	65	Schizophrenia (clinical): 35 Healthy controls: 30	Schizphrenia: 33.51 (10.65) Healthy controls: 34.10 (8.10)	Visual search task	Visual, faces and images, supraliminal	Anger or happiness	1200 ms or 2000 ms
^a^Schwartz et al, 2010^65^	USA	72 in experiment 1 and 71 in experiment 2	FEP in experiment 1 (clinical): 48 and 46 in experiment 2 Healthy controls: 24 in experiment 1 and 25 in experiment 2	FEP: 46.7 (9.7) in experiment 1 and 47.4 (9) in experiment 2 Healthy controls: 49.2 (5.3) in experiment 1 and 45.9 (7) in experiment 2	Modified flanker task	Visual, faces, supraliminal	Anger, fear or neutral	1000 ms
Strauss et al, 2008^66^	USA	63	Schizophrenia (clinical): 41 Healthy controls: 22	Schizophrenia: 41.87 (11.49) Healthy controls: 40 (12.1)	Emotional Stroop task	Visual, words, supraliminal	Happiness, sadness, anger, anxiety or neutral	5000 ms
Strauss et al, 2013^67^	USA	61 in experiment 1 and 54 in experiment 2	Schizophrenia/schizoaffective disorder (clinical): 33 in experiment 1 and 30 in experiment 2 Healthy controls: 28 in experiment 1 and 24 in experiment 2	Schizophrenia/schizoaffective disorder: 40.46 in experiment 1 and 43.41 in experiment 2 Healthy controls: 39.8 in experiment 1 and 44 in experiment 2	Emotional attentional blink task	Visual, words, supraliminal	Pleasant, unpleasant or neutral	175 ms
Strauss et al, 2011^68^	USA	59	Schizophrenia/schizoaffective disorder (clinical): 32 Healthy controls: 27	Schizophrenia/schizoaffective disorder: 42.23 (8.88) Healthy controls: 42.26 (9.56)	Exogenous emotional cueing task	Visual, words, supraliminal	Pleasant, unpleasant or neutral	3000 ms
Sullivan et al, 2017^69^	USA	59	Schizophrenia/schizoaffective disorder (clinical): 30 Healthy controls: 29	Schizophrenia/schizoaffective disorder: 38.6 (11.98) Healthy controls: 41.97 (12.94)	Numerical Stroop	Visual, images, supraliminal	Unpleasant (disgust or threat) or neutral	3000 ms
^a^Taylor et al, 2004^70^	UK	24	Schizophrenia (clinical): 12 Healthy controls: 12	Schizophrenia: 43.9 (14.37) Healthy controls: 42.08 (9.7)	Probe detection task	Visual, words, subliminal/supraliminal	Personally salient (negative or positive), emotional (negative or positive)	100 ms, 500 ms or 1500 ms
Waters et al, 2006^71^	Australia	67	Schizophrenia (clinical): 43 Healthy controls: 24	Schizophrenia: 36.73 (8.41) Healthy controls: 34.67 (8.81)	Affective shifting task	Visual, words, supraliminal	Positive or negative	300 ms
^a^Wiffen et al, 2014^25^	UK	67	FEP (clinical): 44 Healthy controls: 23	SZ: 27.5 (6.7) Healthy controls: 28.5 (11.3)	Emotional counting Stroop task	Visual, words, supraliminal	Psychosis, negative or neutral	–

PDI, Peters Delusions Inventory; FEP, first-episode psychosis.

a. The study was included in the meta-analysis. Study exclusions were a result of lack of data necessary to calculate interference index, despite attempts to contact authors to retrieve the data.

Studies included in the review took place in a range of countries, including France, Australia, UK, USA, Germany, Switzerland, China, South Korea and Spain. The sample size ranged from 18 to 174, totalling 1754 participants, 82% of which had a clinical diagnosis. Clinical groups included in the studies were high risk for psychosis (*n* = 1), ultra-high risk for psychosis (*n* = 1), schizophrenia (*n* = 20), delusional disorder (*n* = 4), persecutory delusions (*n* = 1), first-episode psychosis (*n* = 4), chronic psychosis (*n* = 1), paranoid schizophrenia (*n* = 3) and schizoaffective disorder (*n* = 6).

Attention bias was most frequently measured using the emotional Stroop task (*n* = 18) or the attentional probe task (*n* = 6). Other tasks used were the modified flanker’s task (*n* = 2), the emotional oddball task (*n* = 1), the emotion discrimination task (*n* = 1), the continuous flash suppression (*n* = 1), the emotional Egner’s task (*n* = 1), an audio–visual experimental task, the visual search task (*n* = 1), the emotional attentional bias (AB) task (*n* = 1), the affective shifting task (*n* = 1) and the exogenous emotional cueing task (*n* = 1). A complete list of the tasks used in the different studies, together with a brief description of each task, is provided in Appendix 1. Most of the experimental tasks used word content (*n* = 23), but some used faces (*n* = 10) or images (*n* = 4). Moreover, most studies used threatening (*n* = 15), negatively valenced (*n* = 15), positively valenced (*n* = 20), paranoia or psychosis related (*n* = 5), depression-related (*n* = 9) or anger-related stimuli (*n* = 8) in their experimental tasks.

Three studies in the systematic review included subliminal data points (<100 ms), only one of which was included in the meta-analysis. This can be compared to 33 studies exploring supraliminal stimuli (>100 ms) included in the review, 15 of which were included in the meta-analysis. With only one subliminal effect size it was not possible to consider stimulus exposure duration as a variable in our analysis.

For the meta-analysis, content was categorised into six groups: anger, positive (‘positive’, ‘happiness-related’, ‘mania-related’, ‘personally salient positive’), threat (‘threat’, ‘fear’, ‘anxiety’), paranoia (‘psychosis’, ‘paranoia-related’), depression (‘depression-related’, ‘sadness’) and negative (insufficient information available to further specify, or broaden).

Exclusion of studies at the meta-analysis stage is shown using annotations in Table 1, and was a result of essential data being unavailable, despite contacting authors.

### Findings from the quality assessment

Findings from the study quality assessment are presented in full in Supplementary File 2. Overall quality was good across most dimensions. Global ratings revealed 15, 16 and four studies had overall quality of strong (no ‘weak’ components), moderate (one ‘weak’ component) and weak (two ro more ‘weak’ components), respectively.

Many studies (*n* = 26) received a moderate rating on selection bias, reflecting reasonable representativeness of the target populations (‘strong’ *n* = 3; ‘weak’ *n* = 6). Most studies (*n* = 29) received a strong study design rating, having well-described and executed methods. Confounders were identified as uncontrolled in seven studies, leading to ‘weak’ ratings, but 18 and ten studies received ‘moderate’ and ‘strong’ ratings, respectively. Blinding status on all studies was adequate. The majority (*n* = 32) received a moderate rating with either blinding in place or blinding status assessed as unlikely to affect data quality (e.g. relevant data were from baseline only in a randomised trial). For data collection methods, all except one study was rated ‘strong’ with tools that were both valid and reliable. Completion rates were good across most studies, with all except one receiving moderate (60–79% completion, *n* = 30) or strong (≥80% completion, *n* = 4) ratings.

### Meta-analysis

All robust variance estimation meta-regression models included 14 research teams (clusters) with a total of 35 effect sizes (minimum number of effect sizes in any given cluster = 1, mean number of effect sizes across clusters = 2.5, median number of effect sizes across clusters = 2, maximum number of effect sizes in any given cluster = 8), except for the model in analysis 3, which included 14 studies and 52 effect sizes (minimum number of effect sizes in any given cluster = 2, mean number of effect sizes across clusters = 3.71, median number of effect sizes across clusters = 2.5, maximum number of effect sizes in any given cluster = 12).

**Fig. 2:**
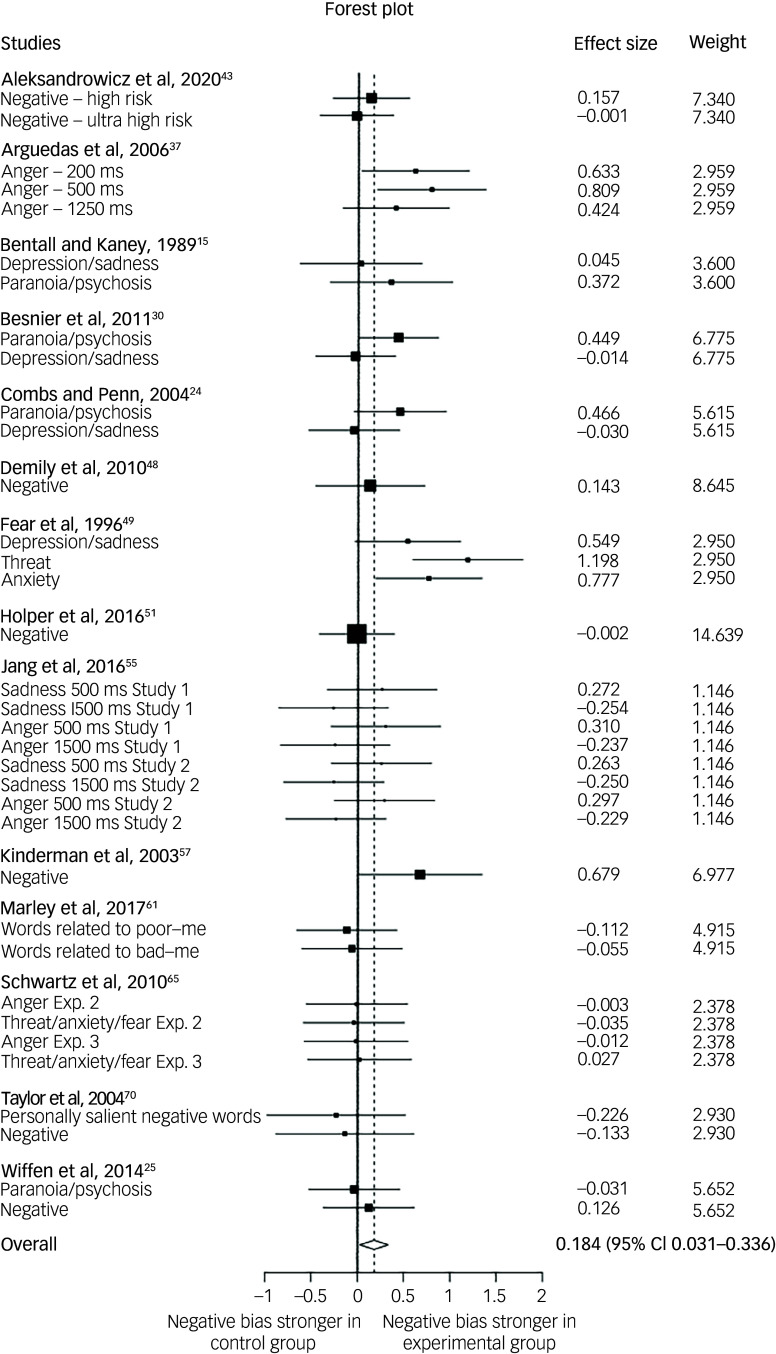
Forrest plot of the attention bias in individuals with paranoia. A black box represents the effect size from an individual study with the size of the box indicates the weight of the study in the meta-analysis. The horizontal lines that extend from the boxes represent the 95% confidence intervals around the effect sizes. The centre of the diamond at the bottom of the plot and the dashed vertical line that comes off it indicate the pooled estimate of attention bias, which appears significant; the 95% confidence interval, reflected in the diamond’s size, does not cross the vertical solid line of no effect. Exp, experiment.

Figure 2 shows a forest plot for the attention bias standardised mean differences across 14 research teams. The raw effect sizes ranged from −0.25 to 1.20, and the pooled effect size was 0.18 (95% CI 0.03−0.33). The between-cluster variance (*τ* ^2^) was 0.03, and the amount of variability in the effect size estimates (*I* ^2^) was 25.40%.

### Analysis 1: Is there a negative attentional bias associated with paranoia?

For analysis 1, the effect size counts for negative stimuli was 35. The average effect size of attention bias across the negative emotional stimuli was 0.26, which was statistically significant (95% CI 0.01−0.52; *p* = 0.046). This suggested that when all the extant eligible data were taken into account, there was an overall small but significant negative attentional bias in experimental paranoia groups compared with controls. The between-cluster variance (*τ* ^2^) was 0.02 and the amount of variability in the effect size estimates (*I* ^2^) was 17.33%. The results from the sensitivity analysis did not alter the inference; the minimum average effect size of attention bias was 0.24 (*p* = 0.044) and the maximum was 0.29 (*p* = 0.027).

### Analysis 2: Does the negative attentional bias differ between clinical and subclinical groups?

For analysis 2, the effect size counts were 29 for clinical status and 6 for subclinical status. The average effect size of negative attentional bias for the clinical samples was 0.25 (95% CI −0.02 to 0.52; *p* = 0.074), and that for the subclinical samples was 0.32 (95% CI −0.01 to 0.65; *p* = 0.059). This suggested that negative attention bias associated with paranoia bias did not, on average, differ significantly from zero for either the clinical or subclinical populations when considered separately. The between-cluster variance (*τ* ^2^) was 0.03 and the amount of variability in the effect size estimates (*I* ^2^) was 22.61%. The minimum and maximum average effect sizes from the sensitivity analysis were 0.22 (*p* = 0.074) and 0.28 (*p* = 0.042), respectively, for the clinical samples, and 0.27 (*p* = 0.125) and 0.46 (*p* = 0.051), respectively, for the subclinical samples.

### Analysis 3: Does the negative attentional bias vary according to stimulus emotional content?

The effects of each category of emotional content of stimuli are presented in Table 2.

**Table 2:** Effect size for each emotional stimulus

Emotional content of stimulus material	Effect size (*N*_ES_)	*p*-value	95% CI
Anger	0.27 (9)	0.247	−0.19 to 0.72
Depression/sadness	0.07 (8)	0.392	−0.10 to 0.24
Negative content	0.07 (10)	0.308	−0.06 to 0.20
Paranoia/psychosis	0.30 (4)	0.027	0.03−0.57
Positive	−0.01 (17)	0.892	−0.11 to 0.09
Threat/anxiety/fear	0.64 (4)	0.180	−0.29 to 1.57

*N*_ES,_ number of effect sizes contributing to this result.

Table 2 shows that only the paranoia/psychosis category was statistically significant, despite the small number of studies contributing. The effect size for this category had a positive value, suggesting that the negative attention bias in the experimental group was 0.30 s.d. higher than that in the control group. The between-cluster variance (*τ* ^2^) and the amount of variability in the effect size estimates (*I* ^2^) were both zero. The sensitivity analyses of re-estimating the pooled effect size after leaving out one effect size at a time did not alter the conclusions, with the exception of the paranoia subtype, where some variation was observed with a minimum and maximum average effect size of 0.23 (*p* = 0.201) and 0.44 (*p* < 0.001), respectively. See Supplementary File 1 for more information.

### Analysis 4: Does the negative attentional bias vary according to type of experimental task used to measure it?

The effect size counts for analysis 4 was 15 for the attentional probe task and 20 for the emotional Stroop task. The average negative attentional bias effect size using the attentional probe task was 0.185, which was not statistically significant (95% CI −0.094 to 0.464; *p* = 0.193). The average effect size using the emotional Stroop task was 0.289, which was also not significant (95% CI −0.01 to 0.59; *p* = 0.060). This suggests that when considered separately, neither task revealed any negative attentional bias that differed statistically significantly from zero. The between-cluster variance (*τ* ^2^) was 0.01 and the amount of variability in the effect size estimates (*I* ^2^) was 20.88%. For the attentional probe task, the results from the sensitivity analysis ranged from 0.15 (*p* = 0.242) to 0.22 (*p* = 0.147), and for the emotional Stroop task, the results ranged from 0.26 (*p* = 0.060) to 0.31 (*p* = 0.039).

### Analysis 5: Does the negative attentional bias differ across faces and word stimuli?

The effect size count for analysis 5 was 15 for faces task and 20 for words. The average negative attentional bias effect size for face stimuli was −0.04, which was not significant (95% CI −0.68 to 0.29; *p* = 0.596). However, the analogous average effect size for words was statistically significant, with a value of 0.41 (95% CI 0.05−0.77; *p* = 0.027). This meant that the difference in negative attentional bias between experimental and control groups was robust and statistically significant when word stimuli were used to measure it. The same could not be said when using face stimuli. The between-cluster variance (*τ* ^2^) was 0.01 and the amount of variability in the effect size estimates (*I* ^2^) was 9.22%. For face stimuli, the results from the sensitivity analysis ranged from −0.10 (*p* = 0.741) to 0.01 (*p* = 0.968), and for words, the results ranged from 0.36 (*p* = 0.031) to 0.45 (*p* = 0.049), which did not alter the inferences made above.

**Fig. 3:**
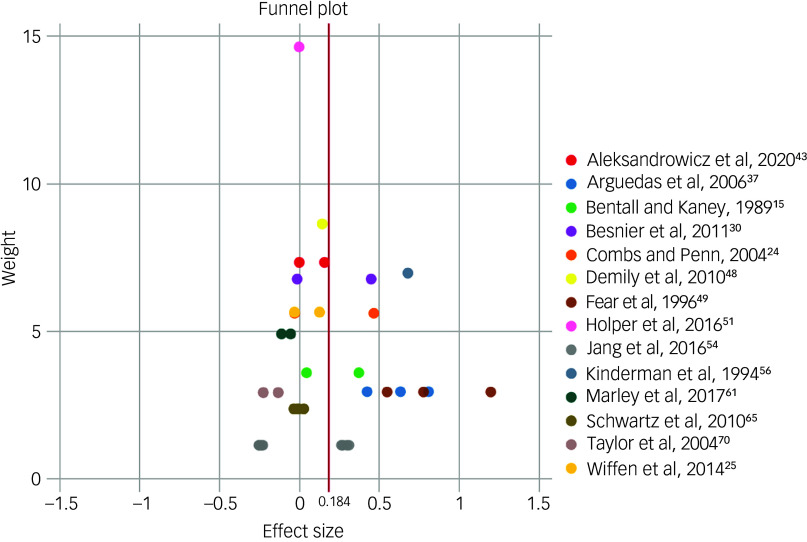
Funnel plot of the studies that were included in the analyses. Studies from the same research team are depicted by the same colour. The vertical red line represents the pooled effect size of attention bias.

Figure 3 shows the funnel plot of the included studies. The *y*-axis measures weight (relative importance of each study included in the meta-analyses) and is calculated on the precision of a study, with larger studies having greater precision and therefore reflecting the true effect size more closely. Smaller studies typically scatter more widely at the bottom as their effect size estimates are less precise. The *y*-axis outlier^51^ is merely a reflection of this study’s very large sample (*n* = 174) compared to the rest of studies. The plot is asymmetrical. The *x*-axis outliers (Fear et al^14^ and Arguedas et al^37^) contribute to a slight positive skew, suggesting the meta-analysis result may have overestimated the true effect size somewhat.

## Discussion

We conducted the first systematic review and meta-analysis on the association between attention bias and paranoia. Our main finding revealed an overall small but significant attentional bias favouring negative over neutral information associated with paranoia. This result is in line with the wider literature showing negative attentional biases in a range of psychopathologies. Our analysis suggests that negative attentional bias is characteristic of individuals with elevated levels of paranoia.

Our second analysis suggested that there were no significance effects of attention bias to negative stimuli in either clinical or subclinical groups. Although our main findings reveal a significant overall effect size for negative attention bias in paranoia, this effect did not remain in either group when the clinical and subclinical subgroups were analysed separately. This could be the result of insufficient power due to the smaller number of effect sizes (and thus smaller sample sizes) within each group. This is a feasible explanation for the subclinical group of studies, with only six studies contributing to the meta-analysis. However, with 29 effect sizes contributing to the clinical group, it is more surprising. The absence of effects in the clinical subgroup may reflect the larger reaction time variability typically seen in clinical populations, which means that subtle reaction time differences, which are typically only tens of milliseconds in selective attentional tasks, may be harder to detect in these samples. It is therefore not possible at present to conclude on whether attentional bias manifests in a dimensional or categorical manner across sub- and full-threshold symptoms.

The current study examined whether content-specific effects reported in other psychopathologies applied in paranoia. Consistent with this, individual studies included in the review revealed a stronger attention bias for paranoia-related stimuli as opposed to other stimuli.^15,46,59^ The meta-analysis findings supported this, showing a significant overall bias effect when stimuli were specifically related to paranoid concerns, but not for other types of stimuli such as anger, fear or general negative material. These results are in line with a systematic review and meta-analysis^29^ from the wider psychopathology literature indicating stronger attention bias when stimuli are disorder-congruent versus disorder-incongruent. Similar content specificity has been found in eating disorders,^72^ chronic pain,^73^ clinical depression^74^ and post-traumatic stress disorder.^75^ However our present findings on content specificity must be treated with caution. Although a significant bias was found when studies used material specifically relevant to paranoia/psychosis, only four effect sizes were available. Indeed, sensitivity analyses showed these effect estimates may not be very stable. Although our results suggest that negative attentional bias is particularly strong when stimulus content closely maps onto paranoid concerns, a larger body of evidence using these materials is needed to be able to draw firm conclusions.

No difference in attention bias related to the type of task used to measure it was found. The apparent contradiction with the main overall finding is likely to be the result of reduced power resulting from smaller numbers of effect sizes within each group contrast. Furthermore, the paucity of studies meant that it was only possible to examine the two most commonly used tasks, the attention probe task and Stroop or Stroop-like tasks. It is possible that with a larger body of evidence across a wider variety of attention tasks, task-related differences may emerge.

Finally, a stronger attention bias was found when word stimuli were used to reveal bias, compared with face stimuli. Again, this finding must be interpreted with caution because of the large imbalance in number of effect sizes available to include at each level of analysis. Studies using word stimuli were far more common than those using faces (11 *v*. 4), which could account for the apparent advantage of words over facial stimuli. One way to redress this going forward would be for researchers to routinely include a condition with face stimuli in their experimental designs, thus allowing a body of work to build up that could address how widely paranoia related attentional bias operates. This would allow us to conclude whether results for paranoia were in line with, or discrepant from, Bar Haim et al’s meta-analysis^5^ sampling anxious individuals, which suggested no differences between naturalistic stimuli and word stimuli in their ability to elicit attentional bias effects. For now, we can conclude that word stimuli can reliably solicit attentional bias in individuals suffering from paranoia.

### Strengths and limitations

A strength of the present study was that we adopted a strong dual-coding methodology throughout, in which every possible stage of study selection, data extraction, coding and bias rating was carried out by two independent raters. This extent of dual coding is relatively rare and provides strong protection against bias, human error and other factors that might compromise the findings. The review protocol was pre-registered on an open-access database (PROSPERO: CRD42022291794).

One strength of this study was the use of raw data, as opposed to reported difference scores, to calculate bias in the same, consistent manner across studies. Another was the use of clustered robust standard errors to correct for dependencies of effect sizes deriving from the same study. Our review revealed heterogeneity of attention bias measures and paradigms, as well as differences in the way bias is defined in different studies, which can make direct comparisons across studies problematic. By using raw condition means to calculate bias independently of authors’ reported values (or, where only bias data were available, checking these were calculated and interpreted correctly) we ensured the best possible validity and equivalence of the effects reviewed.

There are a number of limitations in the present work. One was that three of the studies included in the meta-analysis had no between-participants control group. These studies had only an internal contrast between an emotional and a control stimulus, and had no comparator group of participants without clinical or subclinical psychopathology against which to benchmark the level of attentional bias observed in patients. Most between-participant control groups either show no attentional preference or a slight bias favouring positive/neutral stimuli in preference to negative (ie are biased away from mild negativity). Studies that lack a between participant control condition are therefore likely to slightly underestimate the effect size of negative attentional bias in patients. The inclusion of these three studies may therefore have led to a slight underestimation of the overall negative attentional bias effects associated with paranoia.

There are also inherent limitations in conducting meta-regression analyses, as detailed in Thompson and Higgins. These include confounding and selection of explanatory variables, aggregation bias or ecological fallacy, and lack of statistical power. In this paper we primarily conducted robust meta-regression, which performs best when the Satterthwaite degrees of freedom are greater than four.^75^ However, some of the coefficients in our analyses did not achieve this, with degrees of freedom smaller than four, due to the small number of studies/ effect sizes available for inclusion in the analyses. In this case, the probability of a type one error is larger than the alpha error of 5%, meaning *p*-values should be interpreted with caution. This issue applied in our investigation of content specificity (analysis 3) and in particular with respect to the paranoia-relevant content. The significant attention bias effect for paranoia-relevant stimuli is therefore best interpreted as a series of consistent individual study findings, rather than a single overall effect size. Although we were testing only theory-driven hypothesis, multiple testing may further increase the problems of alpha error inflation.

A further statistical limitation is that, although there is variation in the quality of the studies included in the review, the regression analyses did not include a weighting to account for such difference in quality because of the small number of studies.

Finally, although the moderators we examined in our meta-analysis were derived from relevant previous literature on attention bias in psychological disorders,^5,6^ it remains possible that other important moderating influences may have been missed. Additionally, one known influence on attentional bias – level of conscious awareness of the attended stimuli – could not be included in the analysis because of insufficient relevant data. Therefore, one important recommendation to arise from the current review is that future research involving attention bias in paranoia should routinely include both subliminal and supraliminal experimental conditions.

### Clinical implications

The current review and meta-analysis highlight the important role that negative attentional bias could play in maintaining clinical disorders such as psychosis, where paranoia is frequently a cardinal symptom. Further, by focusing on a single symptom rather than a diagnosis per se, our review has transdiagnostic relevance. Any pathology, or individual presentation, where paranoia symptoms are prominent is likely to be characterised by the negative attentional biases discussed here. Understanding the typical strength and characteristics of this bias can therefore help inform psychological treatment both in general and at the individual level. For example, psychoeducation could now include an explanation of heightened vigilance for negativity that a patient would be likely to experience as a result of attentional bias, and which could, in turn, lead to an exaggerated sense of danger from others and their environment. This is consistent with a cognitive–behavioural approach to psychosis, which is an evidence-based psychological therapy recommended in international clinical guidelines, and which involves developing a shared formulation between therapist and client, highlighting processes such as attentional biases that maintain a sense of threat in paranoia. Our review also has implications for the strengthening of such existing treatments, which could be improved by incorporating elements designed to reduce attentional bias, and the development of new ones, such as attentional versions of cognitive bias modification that specifically target these kinds of biased processes. To date, only cognitive bias modification that is based upon interpretation biases has been adapted to be relevant for paranoia (Hsu et al 2023). The present review suggests that the development of a cognitive bias modification approach using attention bias modification could also be appropriate.

In conclusion, the current systematic review and meta-analysis demonstrated that individuals suffering with paranoia exhibit a small but significant negative attentional bias, similar to that found in other psychopathologies. Although bias appeared strongest for paranoia-related stimuli, and words as opposed to faces, more data is needed to confirm these effects. Our summary of this literature provides a rationale both for existing interventions that target biased attention in paranoia and for the development of new interventions designed to normalise these negative biases.

## Supporting information

Eid et al. supplementary material 1 Eid et al. supplementary material

Eid et al. supplementary material 2 Eid et al. supplementary material

## Supplementary material

The supplementary material is available online at https://doi.org/10.1192/bjo.2026.10993

## Appendix

**Appendix 1:** Description of attentional tasks used in the studies included in the systematic review

Study	Task	Description of the task
Aleksandrowicz et al, 2020^43^	Emotional Stroop task	Participants completed a computerised emotional Stroop task consisting of neutral, positive or negatively valanced words unassociated with psychopathology presented in different colours. Participants were instructed to indicate the name of the colour of the word
*Andersen et al, 2016^44^	Emotional oddball task	Participants completed an emotional oddball task consisting of non-threatening animal (target), neutral, aversive and standard scrambled images. They were instructed to detect the target image among distractors
Arguedas et al, 2006^37^	Emotional attentional probe task	Participants completed an emotional attentional probe task consisting of emotional faces (threat/anger, positive, happiness and neutral emotions) and different stimulus onset asynchrony (of 200 ms, 500 m or 1250 ms) to capture the type of attention bias depicted (engagement or disengagement of attention). The attentional probe appeared either in the same location as the emotional or neutral face. Participants were instructed to indicate its position
*Barbalat et al, 2012^45^	Emotion discrimination task	Participants completed an emotional experimental task consisting of emotional (happy, fearful or angry) and neutral faces. Each trial consisted of instructions for the sequence, eight consecutive faces and a rest period of 2 s. In the initial instructions, participants were asked to follow a rule, which consisted of pressing the red button for a target expression (e.g. press red for angry faces) and green for all other non-target expressions (e.g press green for all non-angry faces)
*Bendall et al, 2014^46^	Emotional Stroop task	Participants completed a computerised emotional Stroop task. The task consisted of neutral or trauma-related words presented in different colours. Participants were instructed to indicate the name of the colour of the written words
Bentall and Kaney, 1989^15^	Emotional Stroop task	Participants completed a card-based emotional Stroop task, which consisted of symbols (Os), neutral, depression and paranoid-related words presented in different colours. Participants were instructed to indicate the name of the colour of the written words
Besnier et al, 2011^30^	Emotional Stroop task	Participants completed a card-based emotional Stroop task, which consisted of symbols (Xs), neutral, depression, and paranoid- and manic-related words presented in different colours. Each card consisted of 50 words. Participants were instructed to indicate the name of the colour of the written words
*Caruana et al, 2011^47^	Continuous flash suppression task	Participants completed a continuous flash suppression task, consisting of two red frames next to each other. Angry and fearful faces (with averted or directed gazes) gradually became visible on the non-dominant eye frame side near a fixation point, while masked stimuli were presented within the non-dominant eye frame. As soon as the face became visible, participants were instructed to indicate the location of the face in relation to a fixation point
Combs and Penn, 2004^24^	Emotional Stroop task	Participants completed the emotional Stroop task, which consisted of paranoia, non-threatening and depression-related words presented in different colours. They were asked to indicate the name of the colour of the written words
Demily et al, 2010^48^	Emotional Stroop task	Participants completed the emotional Stroop task, which consisted of neutral, positive or negative words presented in different colours. They were asked to indicate the name of the colour of the written words
*Fear et al, 1996^49^	Emotional Stroop task	Participants completed a card-based emotional Stroop task, which consisted of symbols (Os), neutral, depression, and threat- and anxiety-related words presented in different colours. Each card consisted of a list of ten rows and five words per row. Participants were instructed to indicate the name of the colour of the written words. Reaction time was measured
Fear et al, 1996^49^	Emotional Stroop task	Participants completed a card-based emotional Stroop task, which consisted of symbols (Os), neutral, depression, and threat- and anxiety-related words presented in different colours. Each card consisted of a list of ten rows and five words per row. Participants were instructed to indicate the name of the colour of the written words. Reaction time was measured
*Feroz et al, 2019^50^	Emotional Stroop task	Participants completed a modified version of the computerised emotional Stroop task, in which each trial comprised two words appearing on the screen; an emotional word in colour (positive, negative or neutral valence) and a colour word in white. The colour of the emotion either matched or did not match the written colour word. Participants were instructed to indicate the name of the colour of the emotional words. Reaction time was measured
Holper et al, 2016^51^	Emotional Stroop task	Participants completed the emotional Stroop task, which consisted of neutral, positive or negative words presented in different colours. They were asked to indicate the name of the colour of the written words. Reaction time was measured
*Hurtado et al, 2018^52^	Egner’s face task – emotional version	Participants completed a modified version of Egner’s face task, which was divided into two tasks. The first one was a non-emotional task in which female and male faces, expressing either fear or happiness, were presented on the screen with the words ‘man’ or ‘woman’ in Spanish written on the face, irrespective of the gender of the face. In the emotional task, the same faces appeared with the words ‘happiness’ or ‘fear’. Participants were asked to indicate the gender of the face in the non-emotional task and the emotion of the face in the emotional task, while ignoring the written words. Reaction times and error rates were recorded
*Ilankovic et al, 2011^53^	Audio–visual experimental task	Participants completed an audio–visual task in which they were recorded reading words from a list. Auditory stimuli consisted of some of the words read in their own voice, some with a distorted self-voice, some with an alien voice and some with an alien distorted voice. Visual stimuli were comprised of the participant’s portraits. Trials began with a fixation cross followed by a cue (portrait). The word was then heard over headphone followed by a response screen where the participants had to indicate if the voice heard was theirs, someone else’s or if they were unsure by using keys on a keyboard. Reaction times were recorded
Jang et al, 2016^54^	Emotional attentional probe task	Participants completed an emotional attentional probe task, which comprised emotional faces (happy, sad, angry). Each trial started with a fixation cross followed by two faces on top of each other. One of the faces was then replaced by an asterisk. Participants were asked to indicate the location of the probe using keys on a keyboard. Auditory feedback (beep) was then presented to participants to indicate is the response was wrong or no response was made. Reaction times were recorded
Jang et al, 2016^55^	Emotional attentional probe task	Participants completed an emotional attentional probe task, which comprised emotional faces (happy, sad, angry). Each trial started with a fixation cross followed by two faces side by side. One of the faces as then replaced by an asterisk. Participants were asked to indicate the location of the probe, using keys on a keyboard. Auditory feedback (beep) was then presented to participants to indicate is the response was wrong or no response was made. Accuracy and latency were measured
Kinderman et al, 1994^56^	Emotional Stroop task	Participants completed a card-based emotional Stroop task, which comprised positive, negative or neutral words presented in different colours. Each card consisted of a list of ten rows and five words per row. Participants were instructed to indicate the name of the colour of the written words. Reaction time was measured
*Kinderman et al, 2003^57^	Emotional Stroop task	Participants completed five emotional Stroop task, which consisted of threatening and neutral words in different colours. Each troop task consisted of one form of threat (sociotropy, autonomy, physical, ego threats perceived to come from others or from self). They were asked to indicate the name of the colour of the written words. Reaction time was measured
*Klewchuk et al, 2007^58^	Emotional Stroop task	Participants completed the emotional Stroop task, which consisted of words related to sexual and physical trauma, negative and positive emotion and neutral words presented in different colours. They were asked to indicate the name of the colour of the written words. Reaction time was measured
*Lim et al, 2011^59^	Emotional Stroop task	Participants completed the emotional Stroop task with threat-related, positive, depression-related and neutral words presented in different colours. They were asked to indicate the name of the colour of the written words. Reaction time was measured
*Liu et al, 2016^60^	Modified Flanker task	Participants completed a modified flanker task. Each trial began with a fixation cross followed by a cue (fearful or neutral face) that would appear on the top or the bottom of the fixation cross. The cue then disappeared, and a row of arrows appeared at either the location previously held by the face or on the other side. The row of arrows consisted of five arrows, four of which were facing the same direction (left or right) and one (the target arrow) facing the other direction. Participants were instructed to indicate the direction of the target arrow by using a joystick. Reaction time was measured
Marley et al, 2017^61^	Emotional Stroop task	Participants completed a attentional probe task, which consisted of neutral words and words related to ‘bad me’ (e.g. evil, nasty, wicked…) or ‘poor me’ (e.g. fake, phony, hollow…) concepts. Word pairings would appear on the screen followed by a attentional probe replacing one of the words on a black screen. Positions of the words and the dots as well as word pairings were counterbalanced. Reaction time was measured
*Moritz et al, 2007^62^	Attentional probe task	Participants completed an emotional task, which consisted of paranoia- or anxiety-related pictures or neutral pictures. Each trial began with a fixation cross in the centre of the screen and two empty boxes on each side of the cross. A picture then briefly replaced of the boxes. A black dot (target) then appeared in one of the boxed. Participants were asked to indicate the location of the target using a button. Reaction time was measured
*Penn et al, 1994^63^	Emotional Stroop task	Participants completed the emotional Stroop task with threat-related words (social threat, social control, physical threat or physical control), colour words and groups of Xs, presented in different colours. They were asked to indicate the name of the colour of the written words. Reaction time was measured
*Pinkham et al, 2014^64^	Visual search task	Participants completed two visual search task. The first task was the ‘face-in-the-crowd-task’ which evaluated social threat by presenting the participants with matrices of nine images that consisted of emotional faces (angry, happy or neutral faces). In that task, angry faces were considered the threatening stimuli. Participants were instructed to determine if the presented stimuli were from the same category or if one of the stimuli was different. The second task was the ‘snake-in-the-grass-task’ and evaluated non-social threats. The latter task was a lightly modified version of the ‘face-in-the-crowd-task’ and consisted of matrices with snakes (threatening stimulus), mushrooms and flowers. Response time and accuracy were measured
Schwartz et al, 2010^65^	Eye gaze task	Participants completed an eye gaze task. Each trial began with a fixation point that was followed by the stimulus (neutral, happy, fearful or angry faces with direct or averted gaze). A word (LEFT or RIGHT) appeared on the face or with an object such as an arrow. Participants were instructed to press a key associated to the word. Reaction time and accuracy were measured
*Strauss et al, 2008^66^	Emotional Stroop task	Participants completed two emotional Stroop tasks: the attention-grabbing task and the lingering effect task. In the attention-grabbing task, participants were asked to name the colour of words presented on the screen. Words were neutral or related to anxiety, sadness, anger or happiness. In the lingering effect task, series of five words were presented instead of single words. The initial word position of the series consisted or a target word (neutral, positive or negative) followed by four neutral words in the remaining four positions. Reaction time was measured
*Strauss et al, 2013^67^	Emotional attention blink task	Participants completed the emotional attention blink task, which consisted of a stream of 15 words (pleasant, unpleasant or neutral), presented one at a time on a screen. Two of the words are target words presented in green on a grey background at two separate times during the stream and the remaining 13 items are distractors and written in black on a grey background. The participants were asked to indicate what the first and second green word were for each stream. In the first experiment, the first target word (T1) was neutral, and the second target word (T2) was emotional. In the second experiment, the first target word (T1) was emotional, and the second target word (T2) was neutral. Accuracy was measured
*Strauss et al, 2011^68^	Emotional cueing task	Participants completed an exogenous emotional cueing task. Each trial began with a fixation point placed in a box on a screen, which was replaced by a word (pleasant, unpleasant and neutral). A target stimulus (letter S or K) then appeared on one side of the box (top, bottom, left or right) for 5 ms before disappearing, and participants were instructed to indicate the location of the target stimulus by pressing a button. Accuracy and reaction time was measured
*Sullivan et al, 2017^69^	Numerical Stroop task	Participants completed a numerical Stroop task. Each trail began with a fixation cross followed by an instruction (‘increase’, ‘decrease’, ‘view’ or ‘watch’). The screen was then replaced with an unpleasant or neutral image followed by a numerical Stroop trial. Participants were asked to indicate the number of digits that were presented on the trial using a video game controller. When presented with the instructions ‘increase’ or ‘decrease’ participants were told to distance themselves from the image and observe it from an external point of view. When presented with the instructions ‘view’ or ‘watch’, they were asked to observe the picture without engaging with any thoughts or feelings. Reaction time was measured
Taylor et al, 2004^70^	Emotional attentional probe task	Participants completed a probe detection task in which word pairs (one neutral word and one personally salient word) were presented on a screen on top of each other. A attentional probe then appeared on the screen in either the location or the top or bottom word. Participants are instructed to press the bar as soon as they detect the dot. Probe detection latencies were reported
*Waters et al, 2006^71^	Affective shifting task	Participants completed the affective shifting task in which they were presented positively or negatively valenced words on a screen. They were asked to press the space bar when detecting target words only. Each block was assigned a target (positive or negative words), which was shifted every two blocks. Reaction time was measured
Wiffen et al, 2014^25^	Emotional counting Stroop task	Participants completed the emotional counting Stroop task, which consisted of five conditions: neutral, psychosis, negative, congruent or incongruent numerical. They were asked to indicate the number of words presented. Reaction time was measured

Note: An asterisk indicates that the study was excluded from the meta-analysis because of a lack of data necessary to calculate interference index, despite attempts to contact authors to retrieve the data.
