# Supplementary material for: Attentional bias in paranoia: systematic review and meta-analysis
Source: BJPsych Open. 2026 Apr 6;12(3):e100. doi: 10.1192/bjo.2026.10993 (PMC13107294; doi:10.1192/bjo.2026.10993)
Supplement: Eid et al. supplementary material 1 — Eid et al. supplementary material [file S2056472426109934sup001.docx]

**Metaregression**

The Stata v.17 command *robumeta* was used with a random-effects weighting scheme and corrections for the residuals and the degrees of freedom due to the small number of clusters in the analyses (Tipton, 2015). We assumed a within-study correlation value of 0.5 after trying out correlation values of 0, 0.2, 0.4, 0.6, 0.8 and 1 and failing to notice any substantial difference in the results. Effect sizes were initially computed as Cohen’s d by subtracting the attention bias mean score of the control group from the attention bias mean score of the experimental group and dividing by the estimated pooled standard deviation of the two groups.

Hedge’s g small sample size corrections were applied using the formula $g=\left( 1-3/(4n-9) \right)d$, where n is the total sample size calculated as the sum of the sample sizes of the two groups, and d is the Cohen’s d. For the sampling variance of the effect sizes, we used the squared estimate of the pooled standard deviation.

**Moderator analyses**

Moderators included in the analysis were coded as follows: type of stimulus presentation (a binary variable coded 0 for face stimuli and 1 for word stimuli), experimental paradigm (0 for “Attentional probe task” and 1 for “Emotional Stroop task”), clinical status (0 for the clinical population and 1 for the sub-clinical population) and content specificity of stimulus (a multinomial variable coded 0 for “Anger”, 1 for “Depression/Sadness”, 2 for “Negative content”, 3 for “Paranoia/Psychosis”, 4 for “Positive content”, and 5 for “Threat/Anxiety/Fear”).

We used Stata’s post-estimation command lincom to report results as linear combinations of the moderator variables. Multinomial moderators entered the models as dummy variables. The I^2 was obtained after re-running the models in R using the package robumeta.

**Forest and funnel plots**

The forest plot was produced using the forest.robu command in R, part of the robumeta package, after regressing the attention bias standardised mean differences on an intercept. The overall effect size displayed in the forest plot is an unadjusted average of the raw Hedge’s g SMDs. The weights and the effect sizes that are reported on the forest plot were then plotted against each other to create a funnel plot and visually inspect the potential for publication bias.

**Individual analyses**

**Analysis 1**. The robust meta-regression model regressed the Hedge’s g SMDs on four dummy variables that were created based on the Content Specificity of Stimulus (CSS) moderator categories, taking the value 1 if the condition was present and 0 if the condition was absent. The dummy variables were: 1) Depression/Sadness, 2) Negative Content (referring to emotional valence of the stimuli, as defined by the authors), 3) Paranoia/Psychosis, and 4) Threat/Anxiety/Fear. For Analysis One, we report the average over the five (four dummies and a constant) estimated coefficients from this model.

**Analysis 2-5**. For the second analysis, we added the binary moderator “clinical status” to the model of Analysis One. This allowed us to calculate two quantities based on the model’s coefficients: an average over the five effects of the negative categories of the CSS moderator for the “Clinical” population, and an average over the five effects of the negative categories of the CSS moderator for the “Sub-clinical” population.

The model for the third analysis resembled that of Analysis One, only this time, the model included, as well as the negative categories, an extra dummy variable for the positive category of the CSS moderator. For this analysis, we report the effect of each one of the CSS categories on attention bias SMD.

For Analysis Four we added the moderator variable “experimental paradigm” to the model of Analysis One and estimated, after running the model, the average over the effects of the negative CSS categories for people who undertook the attentional probe task, and the average over the effects of negative CSS categories for people who undertook the emotional-Stroop task.

Analogously, for Analysis Five, we added the moderator variable “type of stimulus presentation” to the model of Analysis One and estimated an average effect size for “faces” and an average effect size for “words”.

Table S1. Sensitivity analysis results for Analysis 3

| Emotional content of stimulus material | Min. Effect size (p-value) | Max. Effect size (p-value) |
| --- | --- | --- |
| Anger | 0.20 (.268) | 0.30 (.208) |
| Depression/Sadness | -0.01 (.757) | 0.12 (.321) |
| Negative content | 0.02 (.661) | 0.11 (.281) |
| Paranoia/Psychosis | 0.23 (.201) | 0.44 (<.001) |
| Positive | -0.04 (.393) | 0.02 (.756) |
| Threat/Anxiety/Fear | 0.45 (.240) | 0.75 (.070) |
