# Supplementary material for: Attentional bias in paranoia: systematic review and meta-analysis
Source: BJPsych Open. 2026 Apr 6;12(3):e100. doi: 10.1192/bjo.2026.10993 (PMC13107294; doi:10.1192/bjo.2026.10993)
Supplement: Eid et al. supplementary material 2 — Eid et al. supplementary material [file S2056472426109934sup002.docx]

**Study quality assessment results**

**Global ratings**: A weak rating was assigned to studies where more than two components were rated as weak (n= 4), a moderate rating was assigned to studies with one component rated as weak (n= 16) and a strong rating was assigned if the study received no weak rating (n= 15). Ratings are summarized in Table S2.

**Selection bias**: 6 studies received weak rating in this component due to either a lack of representativeness of the target population within the selected sample, less than 60% participation or no information related to selection of the sample is described. 26 studies received a moderate rating as participants somewhat likely represent the target population or 60-79% participated in the study. 3 studies received a strong rating in this component as more that 80% of participants participated in the study and are likely to represent the target population

**Study design**: controlled clinical trials or randomized control trials (n= 29) were associated with a strong rating. A weak rating was received if there was a lack of description of the method used.

**Confounders**: 7 studies were rated as weak as confounders were identified and less than 60% were controlled. 18 studies received a moderate rating if confounders were identified but most have been controlled. 10 studies were rated as strong in this component if there were no confounders identified or most (80%) were controlled.

**Blinding**: No studies received a weak rating which is allocated when researchers were aware of the group allocation and/or participants aware of the research question. 33 studies received a moderate rating if the participant or researcher was not blinded to the intervention, however, there is no indication that the blinding status would affect the quality if the attention bias task was given at baseline. 2 studies were rated as strong if both participants or assessors were blinded or if it was not relevant to the study.

**Data collection methods**: One study received a weak rating as tools were not valid or reliable or not described. No studies received a moderate rating which was allocated when either validity or reliability of the data collection tool was low or missing. 34 studies received a strong rating as tools were both valid and reliable.

**Withdrawal and dropouts**: One study was rated as weak as attrition rate was not described. 30 studies received a moderate rating as 60-79% of participants completed the study and 4 studies received a strong rating as more than 80% of participants completed the study.

**Table S2.** Summary of the Effective Public Health Practice Project (EPHPP) ratings of studies included in the Systematic Review

| Study ID | Selection bias | Study design | Confounders | Blinding | Data collection methods | Withdrawals and dropouts | Global rating |
| --- | --- | --- | --- | --- | --- | --- | --- |
| Aleksandro-wicz 2020 | S | M | M | M | S | S | S |
| Andersen 2016 | M | S | W | M | S | M | M |
| Arguedas 2006 | W | S | M | M | S | M | M |
| Barbalat 2012 | W | M | M | M | S | M | M |
| Bendall 2014 | S | S | W | M | S | M | M |
| Bentall 1989 | M | S | S | M | S | W | M |
| Besnier 2011 | M | M | M | M | S | M | S |
| Caruana 2021 | M | S | M | M | S | M | S |
| Combs 2004 | W | M | W | S | S | M | W |
| Demily 2010 | M | S | S | M | S | M | S |
| Fear 1996 | M | S | M | M | S | S | S |
| Fear 1996 | M | S | S | M | S | M | S |
| Feroz 2019 | M | S | S | M | S | W | M |
| Holper 2016 | W | S | S | M | S | M | M |
| Hurtado 2018 | M | S | S | M | S | W | M |
| Ilankovic 2011 | M | S | M | M | S | W | M |
| Jang 2016 | M | S | M | M | S | S | S |
| Jang 2016 | M | S | M | M | S | W | M |
| Kinderman 1994 | W | M | M | M | S | W | W |
| Kinderman 2003 | W | S | S | M | S | M | M |
| Klewchuk 2007 | M | M | M | M | S | W | M |
| Lim 2011 | M | S | W | M | S | M | M |
| Liu 2016 | M | S | M | M | S | M | S |
| Marley 2017 | M | S | M | M | S | W | M |
| Moritz 2007 | S | S | S | M | S | M | S |
| Penn 1994 | M | S | W | M | S | W | W |
| Pinkham 2014 | M | S | S | M | S | M | S |
| Schwartz 2010 | M | S | W | M | S | M | M |
| Strauss 2008 | M | S | M | M | S | S | S |
| Strauss 2011 | M | S | M | M | S | M | S |
| Strauss 2013 | M | S | M | M | S | M | S |
| Sullivan 2017 | M | S | M | M | S | W | M |
| Taylor 2004 | M | S | W | M | W | M | W |
| Waters 2006 | M | S | M | M | S | M | S |
| Wiffen 2014 | M | S | S | S | S | M | S |

Note: S= Strong rating, M=Moderate rating, W=Weak rating
